# Supplementary material for: Physical Activity Patterns of Women with a Twin Pregnancy—A Cross-Sectional Study
Source: Int J Environ Res Public Health. 2021 Jul 21;18(15):7724. doi: 10.3390/ijerph18157724 (PMC8345556; doi:10.3390/ijerph18157724)
Supplement: Supplementary file 1 [file ijerph-18-07724-s001.zip › ijerph-1278693-supplementary.pdf]

Twin pregnancy and activity survey:

How old are you?

<20

21-30

31-40

41-50

> 51

Where do you live?

Countryside

City < 50.000 citizens

City 50.000-100.000 citizens

City 100.000-500.000 citizens

City > 500.000 citizens

What is your education?

Basic

Secondary

High

Vocational

What was the week of pregnancy at delivery?

Was your last pregnancy twin?

Twin: monochorionic or dichorionic

How tall are you? (cm)

How much did you weight just before pregnancy? (kilograms)

How many kilograms did you put during last pregnancy? (kg) (if you lost weight, you can write for example -4kg)

What was the newborns weight at delivery? (your last pregnancy) (grams)

How many times did you deliver a baby ?

Did the gynecologist inform you about the benefits of exercising during pregnancy?

Yes

No

I do not know

Did the gynecologist inform you about the benefits of exercising during pregnancy?

Yes

No

I do not know

Did you look for information on proper physical activity during pregnancy?
Yes
No
I do not know

Did your gynecologist diagnose some contraindications to aerobic activity during last pregnancy?

We have not talked about physical activity in pregnancy during medical visits
Yes
No

If "yes", what was the contraindication: (only women, who chose yes in the previous question)
Hemodynamically significant heart disease
Restrictive lung disease
Incompetent cervix or cerclage
Multiple gestation at risk of premature labor
Persistent second- or third-trimester bleeding
Placenta previa after 26 weeks of gestation
Premature labor during the current pregnancy
Ruptured membranes
Preeclampsia or pregnancy-induced hypertension
Severe anemia
Hemodynamically significant heart disease

Did you participate in childbirth school classes during pregnancy?
Yes
No

Did the childbirth school provide information on physical activity during pregnancy?
Yes
No
I do not know

Did the childbirth school ensure physical activity classes (e.g. Ball exercises, stretching, etc.)
Yes
No
I do not know

Did you look for information on proper physical activiyty during pregnany?
Yes
No

If yes, where did you look for it?
on the internet
in books

in childbirth school meetings
from friends
from physiotherapist
from midwives
I did not find any reliable source of information.

Did you exercise during your last pregnancy?
(did you perform exercises such as: regular walks, marching, jogging, total body workout at a gym, swimming, yoga, pilates, fitness, exercise-ball workouts or home gymnastics. Exercises had to be done regularly (at least twice a week) and one training should last at least 15 minutes.)
Yes
No

Why you didn’t exercise during pregnancy?
Fear for infant's health
Medical contraindications confirmed by a gynecologist
I didn't see the benefits of pregnancy exercise
Lack of knowledge about benefits of exercise during pregnancy
Because i didn’t want to
Lack of power
Lack of time
Fear for my health
Fear for social opinion

How long did you exercise regularly before pregnancy? (1 session should last at least 15 minutes, exercises had to be done at least 2 times per week)
I did not exercise before pregnancy
Less than half year
Half year
Year
Two years
More than two years

Did you take drugs because of lower the blood pressure, prescribed by a doctor?
Yes
No
I do not know

Did you have preeclampsia during your last pregnancy?
(a condition in pregnancy characterized by high blood pressure (>140/90 mm Hg) accompanied by proteinuria, renal or liver insufficiency or fetal growth restriction)
Yes
No
I do not know

Were you advised to take ursodeoxycholic acid during your last pregnancy by a doctor? (Proursan, Ursopol, Ursocam, Ursofalk, Ursoxyn)
Yes
No
I do not know

Were you advised by a doctor to implement diet with reduced carbohydrates due to abnormal fasting glucose result?
Yes, I was advised with diet
Yes, I was advised with insulin and diet
No

During your last pregnancy, did you have:
Constipation
Lower limbs oedema
Spine ache
Mood swings
Fatigue
Problems with sleeping
Reduction of libido
Heartburn
Calf cramps
Varicose veins
Any ailments

How did you deliver your twins?
Vaginal delivery
Cesarean section
Vaginal delivery first twin and then cesarean section

Did your labour start spontaneously, or it was induced?
Spontaneous
Induced
I do not know

What kind of exercises did you perform during 1 trimesters of pregnancy? (the same question was created for 2 and 3 trimester)
I didn’t exercise in 1,2 or 3 trimesters
Walking
Marching/jogging
Running
Home gymnastics
Exercise-ball workouts/home gymnastics
Total body workout at a gym
Swimming
Yoga
Fitness

Pilates

How many times weekly did you exercise in 1 trimester? (the same question was created for 2 and 3 trimester)
2 times per week
3 times per week
4 times per week
5 times per week
6 times per week
7 times per week
More than 7 times weekly

How much time weekly did you spend on exercising in 1 trimester? (the same question was created for 2 and 3 trimester)
0-60 minutes
60-150 minutes
More than 150 minutes

How long did you exercise during pregnancy?
Till confirmation of pregnancy by a doctor
Till 1 trimester
Till 2 trimester
Till 3 trimester
I stopped 1 week before delivery
I exercised till the end of pregnancy

What did you perform exercises for?
to improve the overall fitness
continue pre-pregnancy physical activity
enable faster recovery after the forthcoming delivery
to prepare yourself for delivery
to help the babies
to sleep better
to have less ailments during pregnancy

Did you have family and partner acceptance in performing physical activity during pregnancy?
Yes
No
I don’t know

Did your exercises meet with a positive reaction from the others?
Yes
No
I don’t know

Did you feel discriminated by social opinion on exercising during a pregnancy?

Yes
No
I don’t know

Did you perform exercises away from home to avoid being recognized or social discrimination?
Yes
No
I don’t know
